# The Next Issue

**Published:** 1868-01-01

**Authors:** 


THE next number of the JOURNAL will be issued on the 20th inst.; thereafter, promptly on the 1st and 15th of each month. It will be mailed hereafter exclusively from the Editor's office.

